# A. V. M. A.

**Published:** 1900-03

**Authors:** 


### SOCIETY PROCEEDINGS.

### A. V. M. A.

SECRETARY STEWART furnishes the following interesting data relative to Association affairs and the coming meeting at Detroit:

President Leonard Pearson has appointed as Committee on Local Arrangements for the annual meeting to be held in Detroit next September, Drs. S. Brenton, J. Hawkins, and G. W. Dunphy. The *personnel* of this committee is an assurance that nothing will be left undone which would contribute to the convenience and pleasure of all in attendance at the meeting.

The several standing committees are actively at work, and the resident secretaries are entering on a vigorous canvass of their respective territories. With all the officers actively at work in the interests of the Association and the coming meeting, coupled with an unusual interest manifested by the membership in general, the indications are most favorable for a large attendance and a grand good meeting in Detroit.

Three papers have already been offered for the literary programme, and the Secretary hopes that others will promptly decide to prepare papers for this meeting, and will promptly notify him. Offers to demonstrate surgical operations are in order, and it is hoped that many will be willing to contribute to this most interesting phase of Association work.

An unusually large proportion of the papers announced in the programme for the New York meeting were actually prepared and offered for consideration. This factor consumed more time than had been planned for, and the Association did not have the privilege of considering some very valuable papers. As it is probable that a larger proportion of papers will be presented hereafter, it would seem advisable to allot three full days for Association work, and leave for the fourth day the enjoyment of social pleasures which it is customary for local veterinarians to provide. It is hoped that members will utilize the columns of the JOURNAL to offer suggestions as to methods for increasing the value of the meetings of the Association to them. The executive officers of the Association will be glad to learn the wishes of the membership, and will use their best endeavors to obviate the difficulties which presented themselves last year.

